# Differential Relevance of NF-κB and JNK in the Pathophysiology of Hemorrhage/Resususcitation-Induced Liver Injury after Chronic Ethanol Feeding

**DOI:** 10.1371/journal.pone.0137875

**Published:** 2015-09-14

**Authors:** Borna Relja, Roxane Weber, Miriam Maraslioglu, Nils Wagner, Tiziana Borsello, Christian Jobin, Ingo Marzi, Mark Lehnert

**Affiliations:** 1 Department of Trauma, Hand and Reconstructive Surgery, University Hospital Frankfurt, Goethe University, Frankfurt, Germany; 2 Neuronal Death and Neuroprotection Unit, Instituto Di Ricerche Farmacologiche "Mario Negri", Milano, Italy; 3 Department of Medicine, Division of Gastroenterology, Hepatology, and Nutrition, University of Florida, Gainesville, Florida, United States of America; Georgia Regents University, UNITED STATES

## Abstract

**Background:**

Chronic ethanol (EtOH) abuse worsens pathophysiological derangements after hemorrhagic shock and resuscitation (H/R) that induce hepatic injury and strong inflammatory changes *via* JNK and NF-κB activation. Inhibiting JNK with a cell-penetrating, protease-resistant peptide D-JNKI-1 after H/R in mice with healthy livers ameliorated these effects. Here, we studied if JNK inhibition by D-JNKI-1 in chronically EtOH-fed mice after hemorrhagic shock prior to the onset of resuscitation also confers protection.

**Methods:**

Male mice were fed a Lieber-DeCarli diet containing EtOH or an isocaloric control (ctrl) diet for 4 weeks. Animals were hemorrhaged for 90 min (32 ± 2 mm Hg) and randomly received either D-JNKI-1 (11 mg/kg, intraperitoneally, i. p.) or sterile saline as vehicle (veh) immediately before the onset of resuscitation. Sham animals underwent surgical procedures without H/R and were either D-JNKI-1 or veh treated. Two hours after resuscitation, blood samples and liver tissue were harvested.

**Results:**

H/R induced hepatic injury with increased systemic interleukin (IL)-6 levels, and enhanced local gene expression of NF-κB-controlled genes such as intercellular adhesion molecule (ICAM)-1 and matrix metallopeptidase (MMP)9. c-Jun and NF-κB phosphorylation were increased after H/R. These effects were further increased in EtOH-fed mice after H/R. D-JNKI-1 application inhibited the proinflammatory changes and reduced significantly hepatic injury after H/R in ctrl-fed mice. Moreover, D-JNKI-1 reduces in ctrl-fed mice the H/R-induced c-Jun and NF-κB phosphorylation. However, in chronically EtOH-fed mice, JNK inhibition did not prevent the H/R-induced hepatic damage and proinflammatory changes nor c-Jun and NF-κB phosphorylation after H/R.

**Conclusions:**

These results indicate, that JNK inhibition is protective only in not pre-harmed liver after H/R. In contrast, the pronounced H/R-induced liver damage in mice being chronically fed with ethanol cannot be prevented by JNK inhibition after H/R and seems to be under the control of NF-κB.

## Introduction

Trauma is the leading cause of deaths in young patients worldwide, with blood loss as the major contributor to mortality after trauma[1, 2]. Hemorrhagic shock followed by resuscitation (H/R) induce a profound local and systemic proinflammatory response, that is characterized by expression and release of numerous proinflammatory mediators, such as interleukin (IL)-6 and the intercellular adhesion molecule (ICAM)-1 or the activation of immune cells (i.e. polymorphonuclear leukocytes) as well as their accumulation (neutrophils) into tissues including liver[3–6]. These changes after H/R often result in cellular and subsequent organ damage that may lead to multiple organ failure and increased mortality rates[7].

One of key players that is involved in the regulation of hypoxic inflammation after H/R denotes the transcription factor nuclear factor-kappaB (NF-κB)[8]. In its inactive form which is present in the cytosol, NF-κB is mostly composed of the p65 and p50 subunits[9]. Its activation as well as its regulation are controlled by the inhibitor of κB (IκB), which prevents its translocation into the nucleus [10]. Activating stimuli of NF-κB including hypoxia, reactive oxygen species or cytokines trigger the phosphorylation and subsequent proteasomal degradation of IκB, followed by the phosphorylation of p65 and its translocation to the nucleus[11]. Upregulated gene expression of pro-inflammatory mediators such as IL-6 or ICAM-1 but also i.e. matrix metallopeptidase (MMP)9 is closely associated with NF-κB signaling and hepatic injury after H/R[3, 6, 11].

Next to NF-κB, activator protein (AP)-1 is involved in both, the H/R-induced systemic and local, hepatic inflammation[12–14]. C-Jun as an essential component of the transcription factor AP-1, which is controlled by the c-Jun N-terminal kinase JNK, mediates *de novo* gene expression of inflammatory genes[15]. Inhibiting JNK and thereby c-Jun activation by the cell penetrating protease resistant JNK inhibitor D-JNKI-1 blunted hepatic damage and local as well as systemic inflammatory changes after H/R[13, 16].

Ethanol-abuse is associated with nearly 50% of all admissions to emergency departments, and plays a significant role within the setting of trauma, not only as preventable cause of deaths but also as a potent immunomodulator[3, 17, 18]. Numerous harmful pathophysiological changes directly affecting the liver, including steatosis, steatohepatitis, fibrosis or cirrhosis, are closely associated with the chronic ethanol intoxication[19]. These changes occurring in the setting of chronic ethanol-use cause an increased susceptibility to H/R-induced liver injury[20, 21]. The underlying mechanisms involve at least in part increased NF-κB activation resulting in enhanced production of local proinflammatory cytokines, including IL-6 and increased infiltration of the liver with neutrophils, resulting in organ damage[20, 21]. Additionally, JNK activation partly contributes to an enhanced hepatotoxicity after chronic alcohol feeding[22].

Given that both, NF-κB and JNK are involved in the pathophysiology of H/R as well as in chronic ethanol abuse, we studied if JNK inhibition by D-JNKI-1 in a combinatory model of chronic ethanol abuse and H/R confers protection regarding the pathogenesis of hepatic injury and inflammation.

## Material and Methods

### Animals and experimental model

Male *cis*-NF-κB^EGFP^ mice (C57BL/6 background) were kindly provided by Christian Jobin and bred under pathogen-free conditions at Mfd Diagnostics (Wendelstein, Germany). At age of 6–8 weeks (20–25 g weight), mice were delivered and acclimatized for 7 days to our in-house facility. Mice were randomly divided into pairs, and assigned to a 4-week pair-feeding protocol according to Lieber-DeCarli diet (Ssniff Spezialdiaeten, Soest, Germany)[23, 24]. Control pair-fed animals received the equal amount of maltodextrin-supplemented diet (ctrl), in order to warrantee the isocaloric feeding. In alcohol-fed animals, the diet protocol consisted of a gradual increase in the EtOH dose in the liquid diet, beginning with 1.75% (v/v) for 5 days, and increasing to 2.63%, 3.50%, 4.38%, and 6.3% (v/v) as described before[20, 25]. After 4 weeks of feeding, animals were weighted and allocated to hemorrhage and subsequent resuscitation group (H/R) or the control (sham) group[20]. Anaesthesia was performed with isoflurane (1.5%, Forane isoflurane, Abbott, Wiesbaden, Germany), and both femoral arteries were exposed and cannulated with polyethylene tubing (Sims Portex, Hythe, UK). The hemorrhagic shock was induced over 5 min by withdrawing blood into a heparinized syringe (10 U) to a mean arterial blood pressure (MABP) of 32 ± 2 mm Hg. Arterial pressure was monitored *via* the second catheter, and recorded using a blood pressure analyzer (BPA 400, Micro-Med, Louisville, KY, USA). After 90 min mice were resuscitated with 60% of the maximal shed blood volume plus a volume of Ringer’s solution corresponding to 50% of the shed blood volume over 30 min *via* the arterial catheter. After resuscitation, catheters were removed, the vessels were occluded, the incisions were flushed with lidocaine and the wounds were closed. Sham-operated animals underwent the same surgical procedures including catheterization of both femoral arteries but hemorrhage was not carried out. Body temperature was measured with a rectal temperature-sensor and maintained at 37°C throughout the experiment with a warming lamp. Animal protocols were approved by the Veterinary Department of the Regional Council in Darmstadt, Germany.

### Group allocation

Sixty-six mice were divided into 8 groups. Sham groups included 4–8 animals, H/R groups 7–12 animals. 4 groups of sham operated animals underwent surgical procedures, but hemorrhage/resuscitation were not carried out. 4 groups of shock animals underwent hemorrhage followed by resuscitation. In one sham and one shock group, respectively, rats were treated either with sterile saline as vehicle (veh) or D-JNKI-1 peptide (11 mg/kg i. p., kindly provided by Dr. Borsello, Milano[26]) after hemorrhage (D-JNKI-1) but before resuscitation. Group sizes, sham: ctrl_veh, n = 8, ctrl_D-JNKI-1: n = 4, EtOH_veh: n = 8, EtOH_D-JNKI-1: n = 7; H/R: ctrl_veh: n = 10, ctrl_D-JNKI-1: n = 7, EtOH_veh: n = 10, EtOH_D-JNKI-1: n = 12.

### Tissue sampling

Two hours after the end of resuscitation, the animals were re-anesthetized for sacrifice. Portal venous blood was collected. The liver was flushed with normal Ringer`s solution, excised and weighted. For each mouse, the two right dorsal liver lobes were snap-frozen in liquid nitrogen. The remaining liver was flushed with normal saline, then fixed with 10% buffered formalin through the portal vein, embedded in paraffin and subsequently sectioned and stained with hematoxylin-eosin (HE).

### Examination of tissue injury

Plasma was stored at -80°C for later analysis of aspartate aminotransferase (AST), alanine aminotransferase (ALT) and lactate dehydrogenase (LDH) using the Spotchem EZ SP-4430 device (Arkray, Japan). Determination of histological damage was performed by an independent examiner who allocated the hematoxylin-eosin stained liver sections to the various experimental groups in a blinded manner as published[13, 16].

### Quantification of cytokine levels

Plasma cytokine concentrations of IL-6 and TNF-alpha were measured using flow cytometry with FACSCalibur (BD Biosciences, Heidelberg, Germany) and the Mouse IL-6 and TNF-alpha Flex Set with a cytometric bead array according to the manufacturer’s instructions (BD Biosciences).

### Ribonucleic acid (RNA) isolation, quantitative reverse-transcription–polymerase chain reaction (RT-PCR)

Total RNA of snap-frozen liver lobes was isolated using the RNeasy-system (Qiagen, Hilden, Germany) according to the manufacturer’s instructions. The residual amounts of DNA remaining were removed using the RNase-Free DNase Set according to the manufacturer`s instructions (Qiagen, Hilden, Germany). The RNA was stored immediately at -80°C. Quality and amount of the RNA were determined photometrically using the NanoDrop ND-1000 device (NanoDrop Technologies, Wilmington, DE, USA). RNA was subsequently reversely transcribed using the Affinity script QPCR-cDNA synthesis kit (Stratagene, La Jolla, CA, USA) following the manufacturer`s instructions and used for qPCR. To determine the mRNA expression of ICAM-1 and MMP9, qPCR was carried out on a Stratagene MX3005p QPCR system (Stratagene) using gene-specific primers for mouse ICAM1 (NM_010493, UniGene#: Mm.435508, Cat#: PPM03196A) and mouse MMP9 (NM_013599 UniGene#: Mm.4406, Cat#: PPM03661C) purchased from Qiagen (Qiagen, Hilden, Germany). As reference gene, the expression of 18srRNA with mouse 18srRNA (Refseq#: K01364, UniGene#: N/A, Cat#: PPM57735E, Qiagen, Hilden, Germany) was measured. Sequences of these primers are not available. PCR reaction was set up with 1x RT2 SYBR Green/Rox qPCR Master mix (SABiosciences) in a 25 μl volume according to manufacturer`s instructions. A two-step amplification protocol consisting of initial denaturation at 95°C for 10 min followed by 40 cycles with 15 s denaturation at 95°C and 60 s annealing/extension at 60°C was chosen. A melting-curve analysis was applied to control the specificity of amplification products. Relative expression of each target gene mRNA level was then calculated using the comparative threshold-cycle (CT) method (2^_ΔΔCT^ method). In brief, the amount of target mRNA in each sample was normalized to the amount of 18srRNA mRNA to give ΔCT. The relative mRNA expression of target genes is presented as fold increase calculated after normalization to 18srRNA and to the corresponding sham group set as 100%. RT-PCR was performed as described before[27].

### Western blotting for intracellular signalling

Liver tissue was homogenized in lysis buffer at 4°C, followed by centrifugation for 30 min at 4°C at 20.000 g and supernatants were stored at -80°C for later analysis. Lysates (50 μg protein) were separated by electrophoresis on 12% polyacrylamide SDS gels and transferred to nitrocellulose membranes (Amersham-Buchler, Braunschweig, Germany). NF-κB (phospho) was detected using rabbit monoclonal Phospho-NF-κB p65 (Ser536) antibody, and NF-κB using rabbit monoclonal NF-κB p65 antibody, respectively (Cell Signaling, Frankfurt, Germany). c-JUN (phospho) was detected using rabbit monoclonal Phospho-c-Jun (Ser63) antibody, and c-Jun using rabbit monoclonal c-Jun antibody, respectively (Cell Signaling, Frankfurt, Germany). Determination of β-actin with anti-β-actin antibody (Sigma, Taufkirchen, Germany) served as a loading control. Blots were blocked (10% non-fat dry milk in 1 mM Tris, 150 mM NaCl, pH 7.4) for 1 h, incubated 1 h at RT with primary antibody (diluted according to manufacturer`s instructions in blocking buffer with 0.5% tween 20 and 0.5% BSA) and then incubated 1 h with horseradish peroxidase-conjugated secondary antibody (Santa Cruz Biotechnology, Santa Cruz, CA, USA) diluted 1:1000 in blocking buffer with 0.5% tween 20 and 0.5% bovine serum albumin at RT. Proteins were detected with ECL™ western blot detection reagents (GE Healthcare, Munich, Germany). Western blot was performed as described previously[13]. Individual bands were semi-quantified by densitometric measurements and the expression state of phosphorylated c-JUN and NF-κB p65 was calculated as the ratio of phosphorylated and total protein values of densitometry data in per cent as described previously[3, 8]. All samples were analyzed twice by western blot. Representative data is shown.

### Statistical analysis

Differences between groups were determined by one-way analysis of variance (ANOVA) using a multiple comparison procedure (Student-Newman-Keuls *post-hoc*). A p value of less than 0.05 was considered significant. Data are given as mean ± standard error of the mean. All statistical analyses were performed employing GraphPad Prism 5 (Graphpad Software, Inc., San Diego, CA).

## Results

### Chronic ethanol feeding and hemodynamic characteristics of hemorrhage and resuscitation

**Fig 1:**
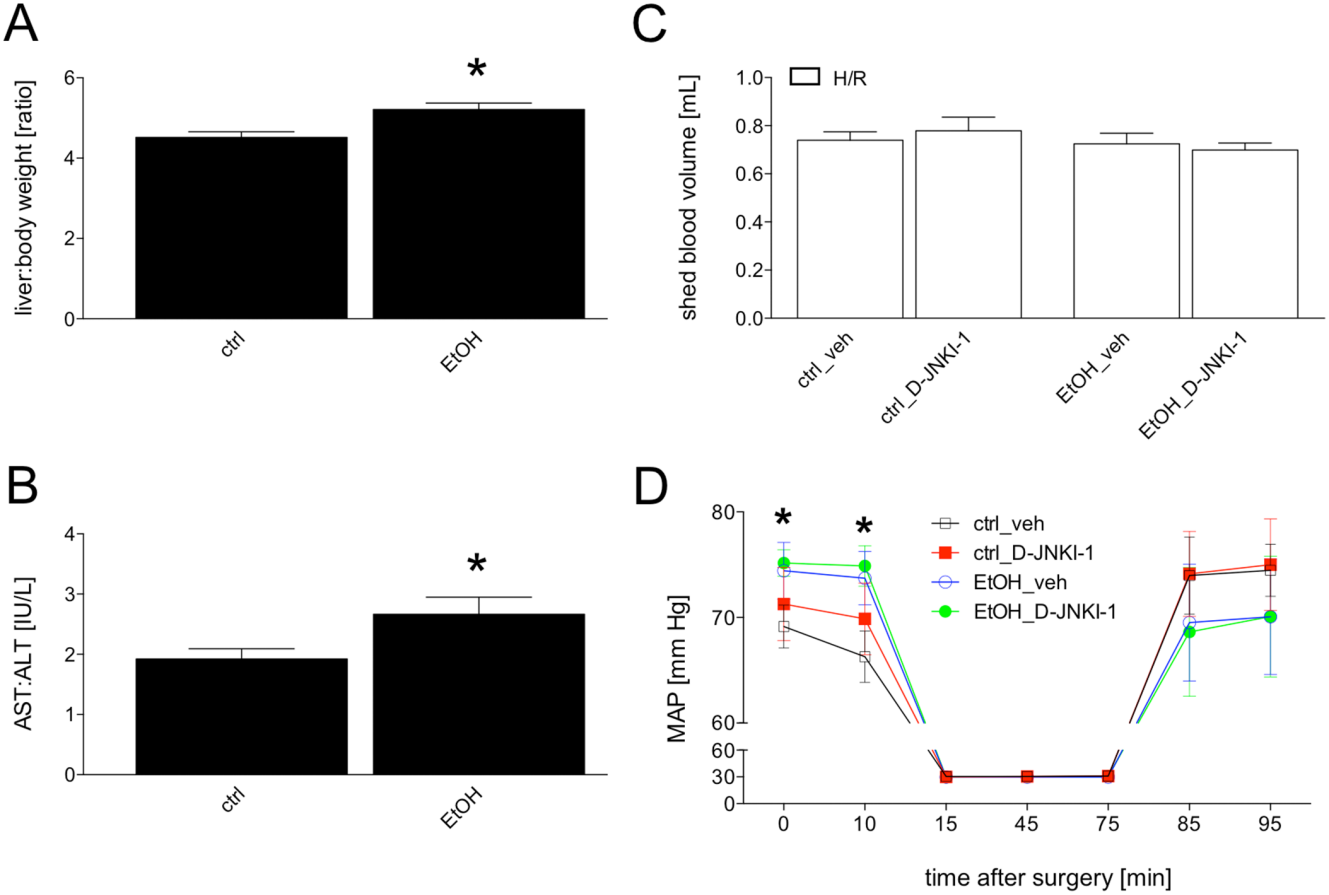
Liver/body weight ratio from pair-fed mice with either ethanol (EtOH, n = 12) or control chow (ctrl, n = 12) is shown (Fig 1A). The ratio of plasma aspartate aminotransferase (AST) and alanine aminotransferase (ALT) is presented (Fig 1B). Fig 1C represents the shed blood volume in mL that was removed for the induction and maintenance of hemorrhagic shock in either ctrl-fed or EtOH-fed vehicle (veh)-treated or D-JNKI-1-treated mice. H/R denotes hemorrhage with subsequent resuscitation. Sham operated animals underwent the same surgical procedures but H/R was not carried out. *: p <0.05 *vs*. ctrl. Fig 1D demonstrates the mean arterial blood pressure (MAP) before and during the hemorrhagic shock as well as during and after the resuscitation (n = 7–12). *: p <0.05 ctrl-veh *vs*. EtOH-veh.

Feeding mice with the ethanol-containing Lieber-DeCarli diet (EtOH) increased significantly the liver/body weight ratio from 4.5 ± 0.1 (n = 12) to 5.2 ± 0.2 (n = 12, p <0.05, Fig 1A). This data indicates an increase in liver weight. As further marker of EtOH-induced hepatic changes, the ratio of systemic AST/ALT was calculated. AST/ALT increased significantly from 1.9 ± 0.2 in the ctrl group (n = 12) to 2.7 ± 0.3 (n = 12) in the EtOH group (p <0.05, Fig 1B).

To evaluate potential effects of both, EtOH as well as the JNK inhibition on the arterial blood pressure in our model, the total shed blood volume that was removed as well as the blood pressures in ctrl or EtOH mice treated with either vehicle (veh) or D-JNKI-1 were measured. During the H/R, blood pressures remained at comparable levels (Fig 1C). EtOH-fed mice had a significantly increased MAP before the H/R procedure compared with pair-fed ctrl group (74.4 ± 2.7 *vs*. 69.2 ± 2.0 mm Hg, p <0.05, Fig 1D). After treatment with either vehicle or D-JNKI-1, blood pressure did not differ between vehicle and D-JNKI-1 treated groups. During the imposition of resuscitation after hemorrhagic shock, the ctrl mice recovered more quickly compared to EtOH-fed mice (Fig 1D), however this was not significant. D-JNKI-1 application did not influence the blood pressure during resuscitation period, and was comparable to vehicle treated mice. The amount of blood removed to induce and maintain hemorrhagic shock at 32 ± 2 mm Hg was comparable in all four groups (Fig 1C). This data suggests that chronic EtOH-feeding increased the MAP. Furthermore, D-JNKI-1 application after completed hemorrhagic shock did not influence the blood pressure either before, during or after resuscitation.

### Cell damage after hemorrhage and resuscitation

**Fig 2:**
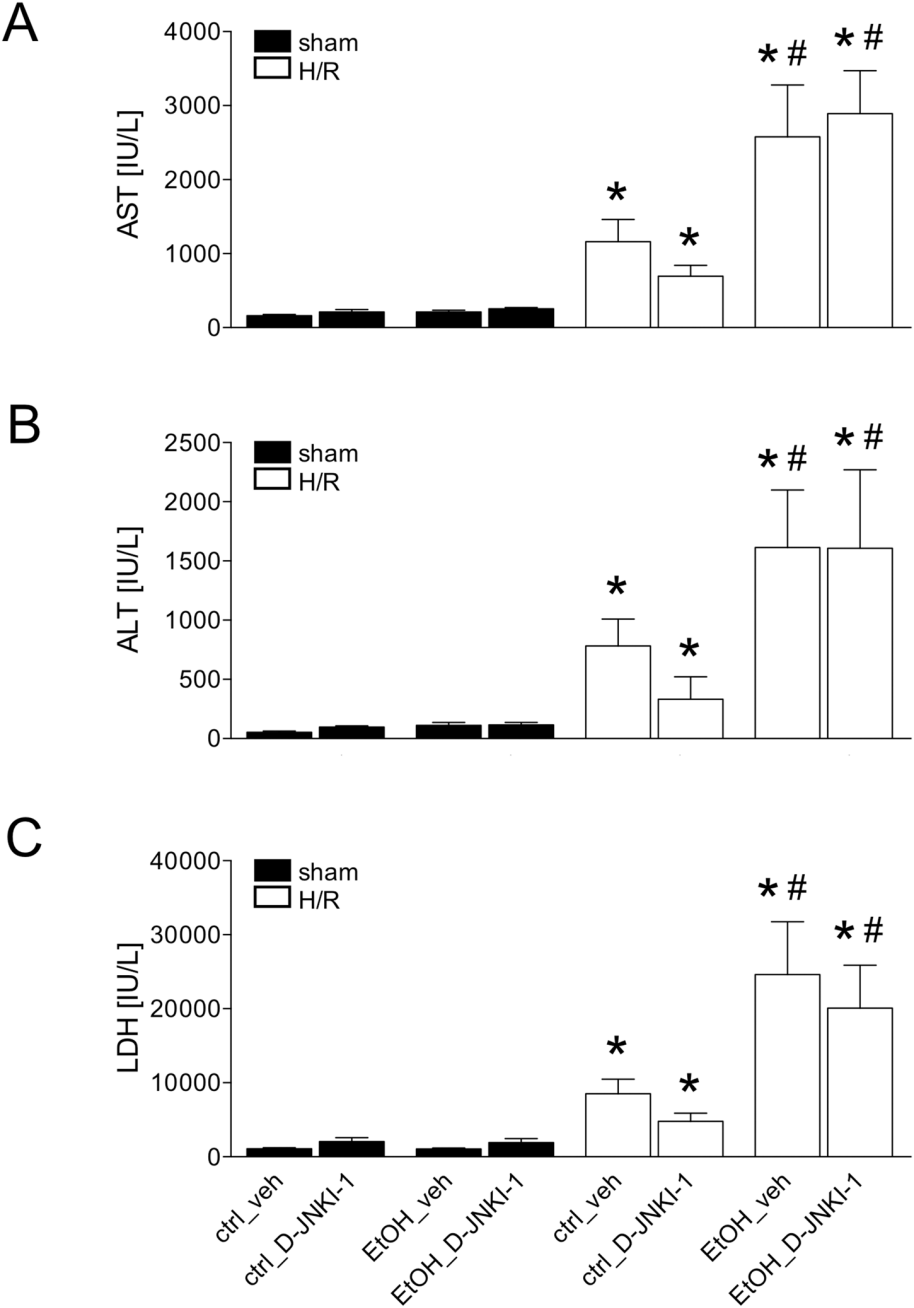
Plasma aspartate aminotransferase (AST, Fig 2A) alanine aminotransferase (Fig 2B) and lactate dehydrogenase (LDH, Fig 2C) levels after pair-feeding with ethanol (EtOH) or control (ctrl) chow. Blood was collected at 2 h after resuscitation for measurement of enzyme levels. H/R denotes hemorrhage with subsequent resuscitation, sham operated animals underwent the same surgical procedures but H/R was not carried out. D-JNKI-1 denotes treatment with the D-JNKI-1 peptide, veh represents vehicle treatment (*: p <0.05 *vs*. corresponding sham group, #: p <0.05 *vs*. corresponding H/R group, sham groups: n = 4–8, H/R groups: n = 7–12).

H/R induced a significant increase of plasma AST to 1162.0 ± 298.2 IU/L at 2 h after resuscitation compared to 162.5 ± 15.2 IU/L after sham operation in vehicle-treated ctrl mice (p <0.05, Fig 2A). H/R-induced AST release was reduced after D-JNKI-1 application by 40% (Fig 2A). In chronic EtOH-fed vehicle-treated mice, H/R-induced AST-release was significantly increased (2577.0 ± 700.0 IU/L) compared with ctrl-fed veh-treated mice (1162.0 ± 298.2 IU/L, p <0.05, Fig 2A). However, in EtOH-fed D-JNKI-1-treated mice, the AST levels (2893.0 ± 577.4 IU/L) were comparable with the AST levels from EtOH-fed vehicle-treated animals (Fig 2A).

H/R induced a significant increase of plasma ALT, a marker of hepatocellular damage at 2 h after resuscitation in ctrl-fed veh-treated mice to 781.9 ± 228.5 IU/L compared to sham operated ctrl-fed veh-treated mice (50.0 ± 11.2, p <0.05, Fig 2B). H/R-induced ALT release was reduced after D-JNKI-1 application by 58% (Fig 2B). EtOH significantly increased the H/R-induced ALT-release (1614.0 ± 483.9 IU/L, p <0.05, Fig 2B). EtOH-induced ALT release after H/R was not changed after D-JNKI-1 treatment (1607.0 ± 663.2 IU/L).

LDH, an indicator of general cell damage raised up to 8529.0 ± 1968.0 IU/L 2 h after resuscitation in the ctrl-fed veh-treated group compared to the sham operated ctrl-fed veh-treated mice (1095.0 ± 113.1, p <0.05, Fig 2C). H/R-induced LDH-release was reduced after D-JNKI-1 application by 44% (Fig 2C). In EtOH-fed veh-treated mice, H/R-induced LDH-release was significantly increased (24605.0 ± 7161.0 IU/L) compared with ctrl-fed veh-treated mice (8529.0 ± 1968.0 IU/L, p <0.05, Fig 2C). In EtOH-fed D-JNKI-1-treated mice, the LDH values (20092.0 ± 5783.0 IU/L) were statistically comparable to EtOH-fed veh-treated mice (Fig 2C).

**Fig 3:**
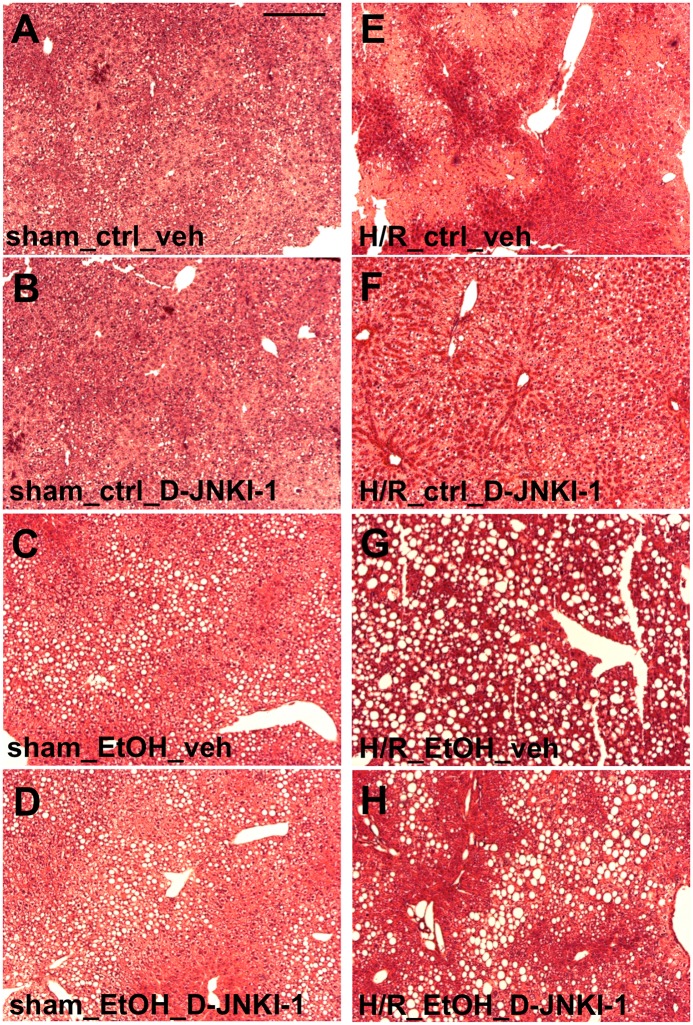
Histological liver necrosis after pair-feeding with ethanol (EtOH) or control (ctrl) chow 2 h after resuscitation. Sham operated animals underwent the same surgical procedures but hemorrhagic shock with resuscitation (H/R) was not carried out. Representative hematoxylin and eosin stained liver lobes from vehicle (veh) or D-JNKI-1-treated mice are shown (Fig 3A: ctrl-fed veh-treated mice, Fig 3B: ctrl-fed D-JNKI-1-treated mice, Fig 3C: EtOH-fed veh-treated mice, Fig 3D: EtOH-fed D-JNKI-1-treated mice after sham operation; Fig 3E: ctrl-fed veh-treated mice, Fig 3F: ctrl-fed D-JNKI-1-treated mice, Fig 3G: EtOH-fed veh-treated mice, Fig 3H: EtOH-fed D-JNKI-1-treated mice after H/R). Bar is 200 μm.

Representative photomicrographs of HE stained liver sections corresponding to data obtained from the measurements of transaminases are shown in Fig 3.

### Systemic pro-inflammatory changes after hemorrhage and resuscitation—plasma IL-6 and TNF-alpha levels

**Fig 4:**
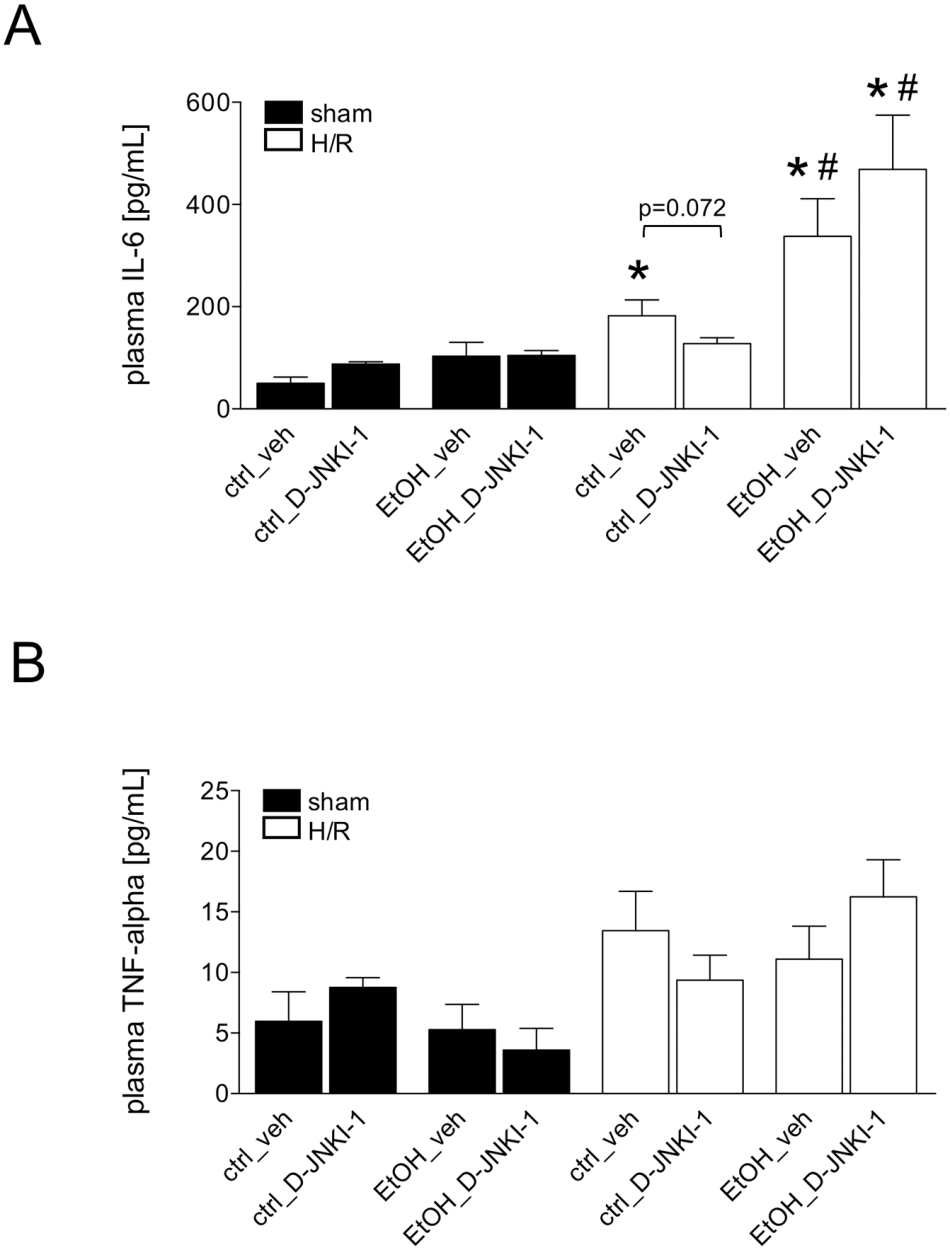
Plasma IL-6 levels (Fig 4A) and TNF-alpha (Fig 4B) levels following hemorrhagic shock and resuscitation (H/R) in pair-fed mice with ethanol (EtOH) or control (ctrl) chow. Sham operated animals underwent the same surgical procedures but H/R was not carried out. D-JNKI-1 denotes treatment with the D-JNKI-1 peptide, veh represents vehicle treatment (*: p <0.05 *vs*. corresponding sham group, #: p <0.05 *vs*. corresponding H/R group, sham groups: n = 4–8, H/R groups: n = 7–12).

Hemorrhage followed by resuscitation induced a systemic immune response, which was determined at 2 h after resuscitation by significantly increased levels of plasma IL-6 in the ctrl-fed veh-treated group compared to the corresponding sham group (182.0 ± 31.3 *vs*. 50.0 ± 12.1 pg/mL, p <0.05, Fig 4A). IL-6 levels were not significantly enhanced in D-JNKI-1-treated ctrl-fed mice compared to the corresponding D-JNKI-1-treated sham group (127.8 ± 11.3 and 87.7 ± 4.6 pg/mL,Fig 4A). However, IL-6 levels trended to a decrease in D-JNKI-1-treated ctrl-fed group after H/R compared with the ctrl-fed veh-treated mice (p = 0.072). The concentrations of IL-6 rose in both, the EtOH-fed veh- as well as D-JNKI-1-treated mice, as compared to H/R-induced IL-6 increase in each corresponding group (EtOH_veh: 337.7 ± 73.6, EtOH_D-JNKI-1: 466.6 ± 106.3 *vs*. ctrl_veh: 182.0 ± 31.3 and ctrl_D-JNKI-1: 127.8 ± 11.3 pg/mL, p <0.05, Fig 4A). The levels for TNF-alpha depicted the similar tendency, but the data was not significant (Fig 4B). These results showed that D-JNKI-1 trended to decrease the systemic pro-inflammatory changes occurring after H/R in ctrl-fed mice, however, in EtOH-fed mice, this trend was not observed in our model.

### Analysis of hepatic gene expression after hemorrhage and resuscitation

**Fig 5:**
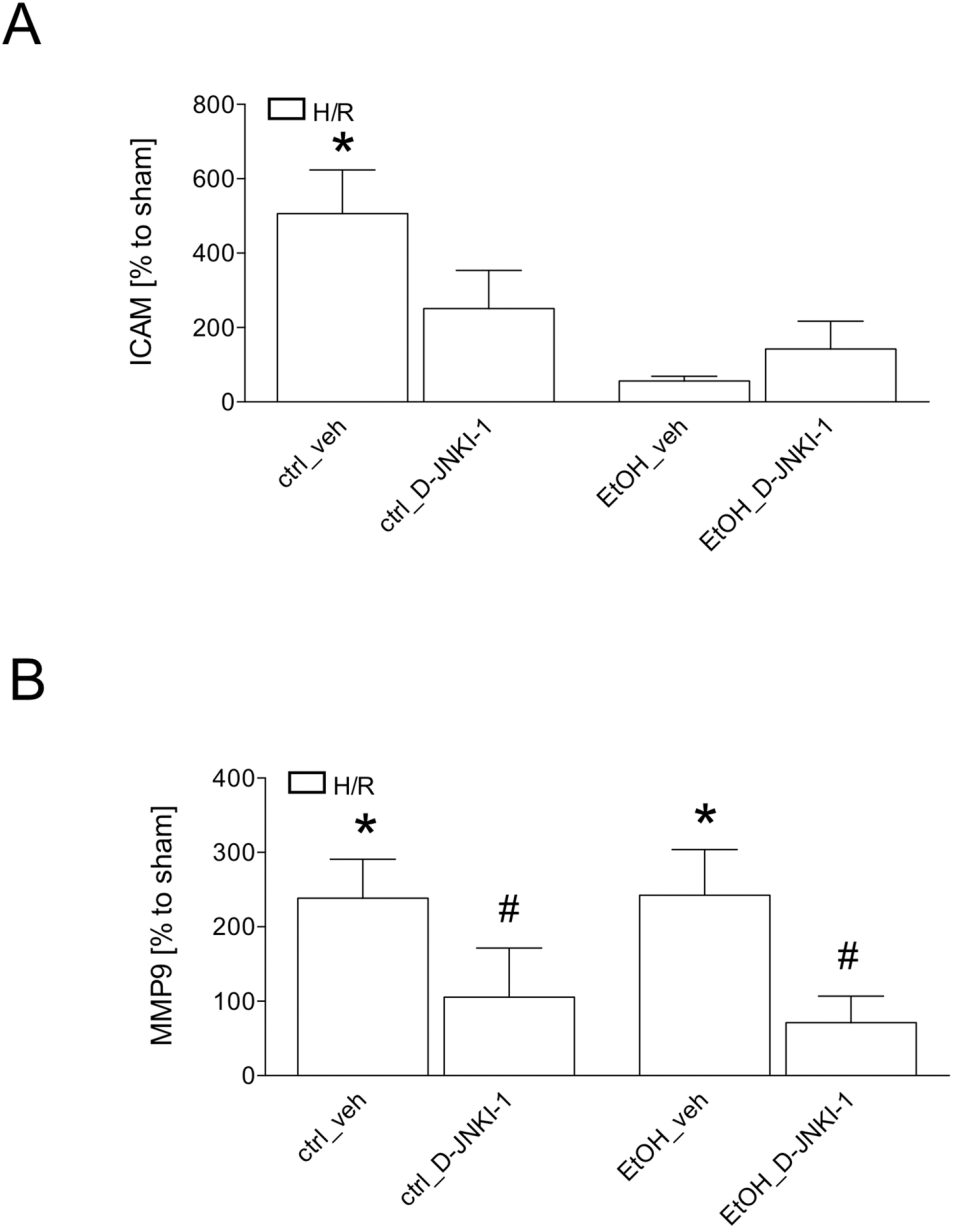
Hepatic ICAM-1 (Fig 5A) and MMP9 (Fig 5B) gene expression at 2 h after resuscitation in pair-fed mice with ethanol (EtOH) or control (ctrl) chow. Sham operated animals underwent the surgical procedures but hemorrhagic shock with resuscitation (H/R) was not carried out. D-JNKI-1 denotes treatment with the D-JNKI-1 peptide, veh represents vehicle treatment. After normalization as described in material and methods, gene expression was measured as % increase compared to 100% of the corresponding sham operated group. (*: p <0.05 *vs*. corresponding sham group, #: p <0.05 *vs*. corresponding H/R group, sham groups: n = 4–8, H/R groups: n = 7–12).

The semi-quantitative real-time PCR showed a significant increase in ICAM-1 gene expression at 2 h after resuscitation in liver samples obtained from ctrl-fed veh-treated mice compared to the corresponding sham group (506% vs. 100%, p <0.05, Fig 5A). This increase was not observed in D-JNKI-1 treated ctrl-fed mice after H/R (251%, Fig 5A). EtOH-feeding trended to reduce ICAM-1 gene expression in mice after H/R, in both veh-treated as well as D-JNKI-1-treated mice, to the levels that were comparable to sham controls (Fig 5A).

MMP9 gene expression were significantly increased after H/R in ctrl-fed veh-treated mice compared to sham controls (238% *vs*. 100%, p <0.05, Fig 5B). This increase in ctrl-fed vehicle-treated mice was significantly reduced by D-JNKI-1 treatment in ctrl-fed mice (105%, p <0.05, Fig 5B). Comparable data was observed in EtOH-fed mice. Here, a strong increase in vehicle-treated mice (243%) compared to the corresponding sham group was significantly reduced by the treatment with D-JNKI-1 (71%, p <0.05, Fig 5B).

### Analysis of the NF-κB and c-JUN phosphorylation after hemorrhagic shock and resuscitation

**Fig 6:**
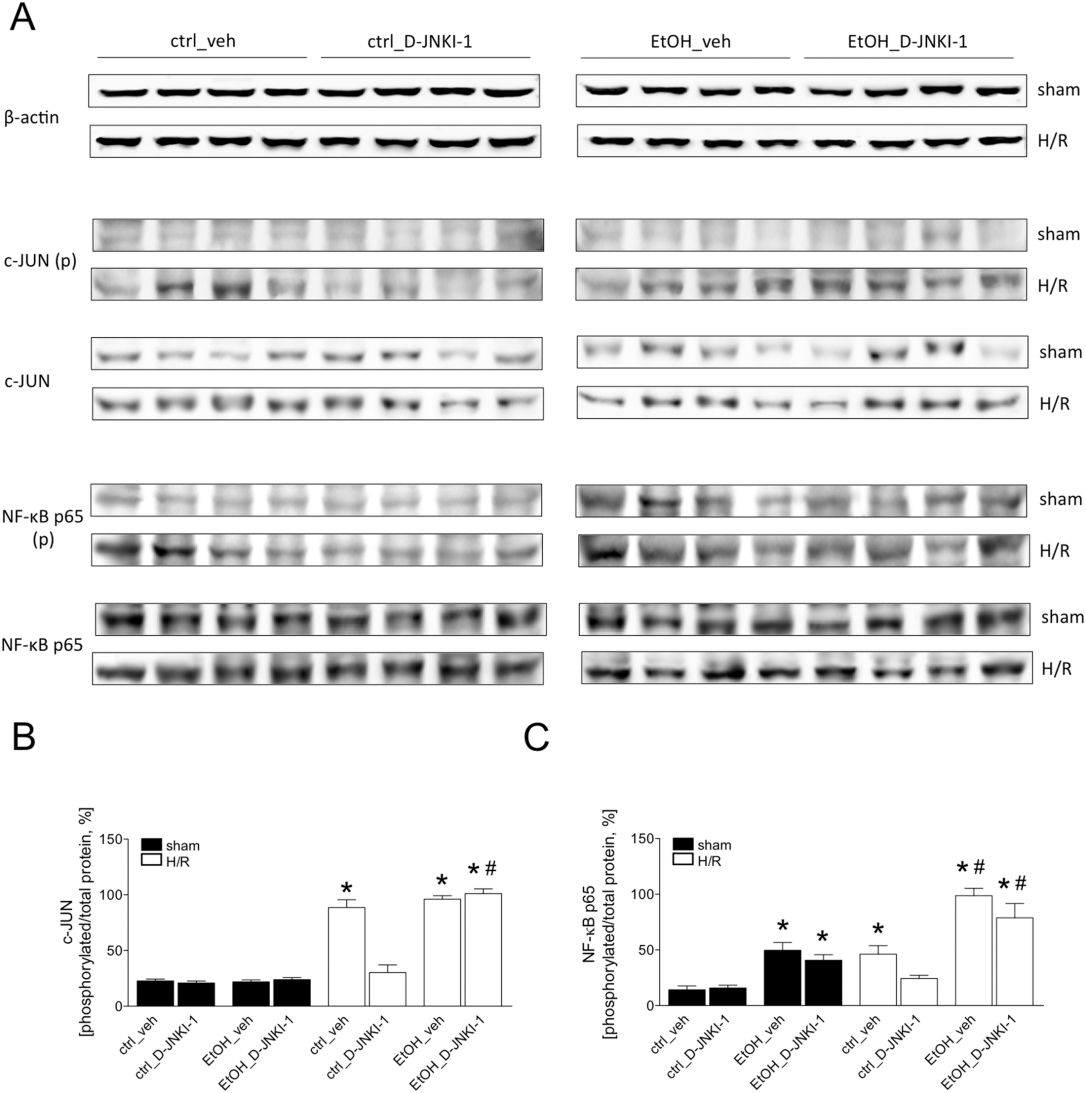
Two h after the end of resuscitation, liver tissue was harvested and western blot for the phosphorylated or non- phosphorylated c-JUN (Fig 6A), p65 subunit of NF-κB and β-actin was performed. Lanes 1–4: liver protein extracts from ctrl-fed mice treated with veh, lanes 5–8: ctrl-fed mice treated with D-JNKI-1, lanes 9–12: EtOH-fed mice treated with veh and lanes 13–16: EtOH-fed mice treated with D-JNKI-1. Sham operated animals underwent the surgical procedures but hemorrhagic shock with resuscitation (H/R) was not carried out. In Fig 6B, the ratio of phosphorylated c-JUN and p65 subunit of c-JUN and NF-κB, respectively, and total protein after densitometric measurements and normalization to β-actin is represented. (*: p <0.05 *vs*. corresponding sham group, #: p <0.05 *vs*. corresponding H/R group).

To evaluate the inhibitory effect of D-JNKI-1 on JNK, the expression levels of phosphorylated c-Jun were analyzed by western blot at 2 h after resuscitation. After H/R, c-Jun phosphorylation was increased in ctrl-fed veh-treated mice compared to the sham group (Fig 6). D-JNKI-1 treatment prevented the increase in the phosphorylation of c-Jun after H/R compared to veh-treated ctrl-fed mice. The transcriptional activity of NF-κB was evaluated by the analysis of NF-κB p65 in liver tissue collected 2 h after resuscitation. Similar to c-JUN phosphorylation, NF-κB p65 phosphorylation was enhanced 2 h after resuscitation in ctrl-fed veh-treated mice compared to the corresponding sham group (Fig 6). The H/R-induced NF-κB p65 phosphorylation was reduced in ctrl-fed D-JNKI-1-treated mice compared to H/R ctrl group.

EtOH-diet did not induce c-Jun, but compared to the ctrl-fed mice or sham, EtOH induced NF-κB p65 phosphorylation at 2 h after resuscitation (Fig 6). D-JNKI-1 treatment did not influence the phosphorylation status of c-Jun and NF-κB p65 in EtOH-fed D-JNKI-1-treated mice. These results demonstrated that H/R activated JNK, thereby resulting in enhanced c-Jun and NF-κB p65 phosphorylation levels. EtOH-feeding increased NF-κB p65 phosphorylation but did not alter c-Jun phosphorylation. This effect in ctrl-fed mice is substantially inhibited after D-JNKI-1 treatment, however, such reduction was not observed in EtOH-fed mice.

## Discussion

In the present study, we have examined the role of specific JNK inhibition on general liver injury and proinflammatory changes induced by hemorrhage followed by resuscitation (H/R) in chronically ethanol (EtOH)-fed mice. Furthermore, our data indicates the important role of NF-κB activation in this model. H/R-induced hepatic injury and increased release of systemic proinflammatory cytokine IL-6 were further enhanced by chronic EtOH abuse. These changes were associated with the phosphorylation of the stress kinase JNK and the transcription factor NF-κB, suggesting that the activation of both, JNK and NF-κB may be involved in the pathogenesis of liver damage induced by H/R in control-fed but also EtOH-fed mice in our model. The specific inhibition of JNK by D-JNKI-1 conferred protection through a JNK and NF-κB-dependent mechanism in not pre-harmed liver, however in chronically EtOH-fed mice JNK inhibition did not prevent the H/R-induced hepatic damage and proinflammatory changes or JNK activation and NF-κB phosphorylation after H/R. Interestingly, c-JUN phosphorylation was not enhanced by EtOH-diet, while EtOH abuse increased the phosphorylation of NF-κB p65.

Alcohol intoxication has detrimental effects on post-injury complications and outcomes in major trauma patients due to immunomodulatory properties of EtOH[28, 29]. Feeding with EtOH-diet according to the model of Lieber-DeCarli that was used for the present study, resulted in hepatic steatosis, a characteristic liver change induced by chronic EtOH ingestion as demonstrated in our previous study[20, 24, 30]. Here, showing the EtOH-induced elevation of the liver/body weight and AST/ALT ratio confirmed that chronic EtOH abuse resulted in steatosis as described before[20]. The immunomodulatory effect of EtOH was expressed in increased IL-6 levels and enhanced activation NF-κB. JNK activation as depicted by c-Jun phosphorylation was not increased by EtOH abuse alone, however JNK was induced by H/R in EtOH-fed animals.

With regard to the liver, JNK and NF-κB are activated after H/R resulting in increased expression and release of inflammatory mediators including IL-6 and liver damage[13, 31, 32]. Consistent with previous findings, we detected enhanced systemic IL-6 levels after H/R and even more profound increase after H/R in EtOH-fed animals[20]. IL-6 KO mice that were protected from hepatic injury demonstrated the importance of IL-6 in H/R-induced pathophysiology of liver injury[33]. Another study demonstrated enhanced systemic IL-6 levels to be associated with the phosphorylation of NF-κB p65 and H/R-induced liver damage[8]. Given the well-described changes in IL-6 after H/R and EtOH abuse, here IL-6 was used to model the inflammatory response in this setting. Combining chronic EtOH abuse with H/R increased even more the JNK and NF-κB activation, promoting thereby enhanced liver damage in this combinatory model.

D-JNKI-1, syn. XG-102 is a highly specific and stable cell permeable inhibitory peptide[34]. After an acute intraperitoneal administration of D-JNKI-1 in mice, it has been detected widely spread in main organs notably in the liver and kidney[34]. D-JNKI-1 derives from linking the 20 amino acids terminal JNK-inhibitory sequence, the JNK-biding domain of the JNK interacting protein-1 (JIP-1)/Islet brain (IB)-1 to a ten amino acids HIV-Tat (48–57) transporter sequence, which enables an active transport of the peptide into cells[35, 36]. Subsequently, the interaction of the JNK with its substrates is blocked. Compared to small chemical inhibitors (SP600125 or AS601245) D-JNKI-1 uses a different inhibitory mechanism. These inhibitors competitively target the ATP binding site not only of JNK but also of other kinases, and therefore, concerns about their specificity have been raised[37, 38]. In several preclinical and clinical settings including numerous degenerative diseases D-JNKI-1 has demonstrated benefit without undesirable side effects[13, 16, 36, 39–44]. However, the mechanisms of D-JNKI-1 are not fully understood. The variation of the length of Tat-sequence and D-stereometry enable enough variations of half-life and high stability[36]. Furthermore, the use of the Tat-cargo peptide is a highly efficient cell-delivery strategy[45, 46] and may provide drug delivery even under pathological conditions. Notably, the fluorescently labelled Tat-D-JNKI-1 peptide inhibitor of JNK accumulates passively in the nuclei of neurons in the cortex within one hour after being i.p. injected into rats[47]. However, specific pharmacokinetic data upon drug delivery, its uptake, the underlying mechanisms and intracellular distribution in the setting of hepatic haemorrhage/resuscitation require further studies.

JNK becomes activated upon a wide variety of inflammatory cytokines but also by the exposure to environmental stress[48]. C-Jun as a target of JNK has been identified to contribute to H/R-induced liver damage, and treating mice with D-JNKI-1 lowered c-Jun activation after H/R. This effect conferred a substantial liver protection and reduction of the inflammatory response as indicated here and reported previously[13]. Based on the current literature possible beneficial effects of treating animals with D-JNKI-1 after H/R that were chronically fed EtOH may be expected and have not been analyzed before. However, due to manifest and detrimental tissue changes after chronic alcohol exposure, the use D-JNKI-1 in this combinatory model may be of only limited potential. In the present study, we observed a hyper-activation of JNK due to increased c-Jun phosphorylation after EtOH feeding combined with H/R. This data suggests, that JNK is at least in part responsible for increased H/R-induced liver damage and proinflammatory changes in EtOH-fed mice. Interestingly, there was no induction of c-JUN phosphorylation by EtOH diet alone. It appears that EtOH-induced liver damage is not JNK dependent. However, D-JNKI-1 was not able to reduce c-JUN phosphorylation induced by H/R in EtOH-fed animals. Possibly, the delivery mechanisms of D-JNKI-1 in alcohol-damaged liver after H/R are not available. Koteish *et al*. have reported that chronic EtOH use potentiates lipopolysaccharide liver injury despite JNK inhibition[49]. In line with these and our findings, no significant changes in JNK activation were found in livers from chronically EtOH-exposed animals[50].

On the other hand, NF-κB as a key regulator of numerous inflammatory mediators such as IL-6 or TNF-alpha is known to be involved in the H/R-induced pathogenesis of liver injury in both, control-fed and EtOH-fed animals[3, 20, 21, 31]. Consistent with these findings, increased hepatic MMP9 expression and NF-κB p65 phosphorylation confirmed the NF-κB activation in both, H/R-induced conditions as well as in combined EtOH and H/R conditions. Previously, we have found that NF-κB inhibition in the setting of H/R suppressed the proinflammatory response and liver damage[6, 32]. Consistent with this data, here, inhibition in the phosphorylation of the p65 subunit was found in D-JNKI-1 treated group after H/R. This was associated with normalized cytokine levels and hepatic histology after H/R. However, in line with previous findings, NF-κB p65 phosphorylation was induced by EtOH-feeding alone in our *in vivo* model of alcohol-induced liver damage[20, 25]. Even though D-JNKI-1 appears to lower the NF-κB activation as shown by slighty reduced p65 phosphorylation and MMP9 expression after H/R in EtOH-fed animals, this effect had no beneficial character in our model after H/R in EtOH-fed animals.

Taken together, JNK inhibition is protective only in not pre-harmed liver after H/R. The pronounced H/R-induced liver damage in mice chronically fed ethanol seems to be rather under the control of NF-κB and was not prevented by JNK inhibition. Additionally, in the present study, only one dose of D-JNKI-1 for the JNK inhibition was applied. Nevertheless, there is compelling evidence that NF-κB activation due to chronical exposure to EtOH minimizes the possible therapeutic options for H/R-induced proinflammatory changes and liver damage.

## Supporting Information

S1 Data Graph data. (XLSX)
